# Less is more: neural mechanisms underlying anomia treatment in chronic aphasic patients

**DOI:** 10.1093/brain/awx234

**Published:** 2017-09-27

**Authors:** Davide Nardo, Rachel Holland, Alexander P Leff, Cathy J Price, Jennifer T Crinion

**Affiliations:** 1 Institute of Cognitive Neuroscience, University College London, London, UK; 2 Division of Language and Communication Science, City University London, London, UK; 3 Department of Brain Repair and Rehabilitation, Institute of Neurology, University College London, UK; 4 Wellcome Trust Centre for Neuroimaging, Institute of Neurology, University College London, London, UK

**Keywords:** aphasia, word retrieval, anomia treatment, phonemic cueing, picture naming

## Abstract

**See Thompson and Woollams (doi:10.1093/brain/awx264) for a scientific commentary on this article**.

Previous research with aphasic patients has shown that picture naming can be facilitated by concurrent phonemic cueing [e.g. initial phoneme(s) of the word that the patient is trying to retrieve], both as an immediate word retrieval technique, and when practiced repeatedly over time as a long-term anomia treatment. Here, to investigate the neural mechanisms supporting word retrieval, we adopted—for the first time—a functional magnetic resonance imaging task using the same naming procedure as it occurs during the anomia treatment process. Before and directly after a 6-week anomia treatment programme, 18 chronic aphasic stroke patients completed our functional magnetic resonance imaging protocol—a picture naming task aided by three different types of phonemic cues (whole words, initial phonemes, final phonemes) and a noise-control condition. Patients completed a naming task based on the training materials, and a more general comprehensive battery of language tests both before and after the anomia treatment, to determine the effectiveness and specificity of the therapy. Our results demonstrate that the anomia treatment was effective and specific to speech production, significantly improving both patients’ naming accuracy and reaction time immediately post-treatment (unstandardized effect size: 29% and 17%, respectively; Cohen’s *d*: 3.45 and 1.83). Longer term gains in naming were maintained 3 months later. Functional imaging results showed that both immediate and long-term facilitation of naming involved a largely overlapping bilateral frontal network including the right anterior insula, inferior frontal and dorsal anterior cingulate cortices, and the left premotor cortex. These areas were associated with a neural priming effect (i.e. reduced blood oxygen level-dependent signal) during both immediate (phonemically-cued versus control-cue conditions), and long-term facilitation of naming (i.e. treated versus untreated items). Of note is that different brain regions were sensitive to different phonemic cue types. Processing of whole word cues was associated with increased activity in the right angular gyrus; whereas partial word cues (initial and final phonemes) recruited the left supplementary motor area, and right anterior insula, inferior frontal cortex, and basal ganglia. The recruitment of multiple and bilateral areas may help explain why phonemic cueing is such a successful behavioural facilitation tool for anomia treatment. Our results have important implications for optimizing current anomia treatment approaches, developing new treatments, and improving speech outcome for aphasic patients.

## Teaser

Naming in aphasia can be facilitated by phonemic cueing, both as an immediate retrieval strategy and as a long-term treatment. Nardo *et al.* report that immediate and long-term facilitation of naming rely on common neural networks, while whole words and partial cues promote naming via different networks.

## Introduction

Anomia—the inability to retrieve words an individual wants to say—is the most common symptom of aphasia post-stroke, often regardless of severity and lesion location (Goodglass, 1993; Crinion and Leff, 2007). The deficit typically persists in chronic aphasia, and constitutes a serious obstacle to patients’ speech, communication and effective functioning in everyday life (Code, 2003; Johansson *et al.*, 2012). As such, anomia and its treatment is seen as a hallmark for aphasia recovery.

Functional imaging studies to date have mainly focused on naming performance as their primary outcome measure for aphasia treatment success (for a review, see Crinion and Leff, 2015). All report significant changes in brain activity following treatment with some degree of consistency. For some, left perilesional activation is deemed critical for aphasia recovery (Fridriksson, 2010; Rochon *et al.*, 2010; Fridriksson *et al.*, 2012; Abel *et al.*, 2014), while others report significant bilateral changes (Fridriksson *et al.*, 2007; Vitali *et al.*, 2007; van Hees *et al.*, 2014; Abel *et al.*, 2015). In the case of speech production, the contribution of language homologue areas in the right hemisphere and the direction of these effects (i.e. increase versus decrease of task-dependent activation) remains hotly debated, particularly with respect to whether right frontal cortices—including Broca’s area homologue—play a beneficial (Blasi *et al.*, 2002; Crinion and Price, 2005) or detrimental (Naeser *et al.*, 2005; Winhuisen *et al.*, 2005) role in recovery. Accompanying these functional brain changes within the language network itself (e.g. left inferior frontal and superior temporal cortices, as in Fridriksson *et al.*, 2012), are reported changes in additional non-language cognitive networks (Fridriksson *et al.*, 2007; van Hees *et al.*, 2014), leading some authors to propose that successful aphasia (particularly speech) treatment requires recruitment of both language and domain-general networks to facilitate recovery (Vitali *et al.*, 2007; Fridriksson, 2010; Rochon *et al.*, 2010; Abel *et al.*, 2014, 2015; Brownsett *et al.*, 2014).

A key factor that could help explain the variability of reported aphasia recovery results and treatment effects is the nature of the therapy administered to drive the brain and behavioural change. Many studies have used confrontation picture naming as their functional MRI task and outcome measure of aphasia treatment. This methodological choice allows the neural correlates underlying the post-therapeutic outcome to be investigated. However, the treatment approaches used have varied widely across studies (e.g. semantic versus phonological, errorful versus errorless, etc.), with a wide range in dose (total hours range from 12 to 56); intensity (hours per week from 5 to 15); and number of items treated (from 30 to 80). When the functional MRI task (e.g. free-naming) is different from the task used in aphasia treatment (e.g. cued-naming, or spoken word-to-picture matching), the interpretation of the imaging results is complicated (i.e. it is not clear how therapy facilitates brain and behavioural change in these patients). To improve aphasic patients’ speech recovery and outcome we need to investigate, understand and optimize the therapeutic mechanisms themselves that are driving the brain and behavioural change. This constitutes the focus of our present study.

A striking feature of anomia is that phonemic cues immediately aid word retrieval in many patients. Patients who are unable to name a given item (e.g. ‘car’) find they can say the word perfectly when given an auditory cue (e.g. the initial phoneme /ka/ or whole word /ka:r/). The cues convey speech sound information about the word in question (Pease and Goodglass, 1978; Kendall *et al.*, 2008). Pairing these cues with pictures of items a patient repeatedly practices naming can result in long-term (un-cued) naming improvement (for reviews see Howard, 1994; Maher and Raymer, 2004), and clinically meaningful speech gains (Raymer *et al.*, 2007; Lambon Ralph *et al.*, 2010; Best *et al.*, 2013). It has been suggested that this phonemic cueing treatment approach relies upon the same processes that underlie priming in unimpaired speakers (Best *et al.*, 2002; Nickels 2002), where phonemic cues prime the retrieval of a word’s correct phonological form (Miceli *et al.*, 1996; Starreveld, 2000). In aphasic patients, the hypothesis is that naming improvements using this treatment approach rely on recruitment and priming of residual ‘normal’ naming neural networks (Madden *et al.*, 2017). However, despite the longstanding use of phonemic cues to aid naming in clinical practice, surprisingly the neural mechanisms underlying this treatment approach have not been investigated to date.

To do this, we designed two complementary experiments. The first experiment (Experiment 1: Free-naming; behavioural only) asked patients to perform a confrontation picture naming task, without the aid of any auditory cues at three time points: before (T1), directly after (T2), and 3 months after (T3) completion of an intensive, high-dose anomia treatment programme using the phonemic cued-naming approach and a large pool of items (see below).

The second experiment (Experiment 2: Cued-naming; conjoint behavioural-functional MRI) focused on the neural mechanisms implicated in word retrieval as it occurred during the therapeutic process at two time points: before (T1) and directly after (T2) the 6-week anomia treatment. Here, patients performed a picture naming task while in the functional MRI scanner, on a selected subset of items (both treated and untreated), presented concurrently with different auditory cue-types: three phonemic cues (whole words, initial phonemes, final phonemes) and one control condition (noise). This enabled us to investigate the neural mechanisms underlying: (i) immediate versus long-term cued facilitation of naming, in order to shed light on their relative nature; but also (ii) naming facilitation by different types of phonemic cues, to clarify whether they rely on shared or different neural processes. To our knowledge, the present study is the first to adopt a functional MRI task that directly mirrors the training task used in an anomia treatment programme. The comparison between cued and control items allowed us to investigate the immediate facilitation of naming, as per what was also happening in each cued-naming treatment session the patients completed. The comparison between treated and untreated items after treatment allowed us to characterize the consolidation of training utilizing these cues and long-term facilitation effects on naming performance.

We predicted that long-term word relearning and recovery would be correlated with increased efficiency and reduced blood oxygen level-dependent (BOLD) signal within the same bilateral residual speech network primed by phonemic cueing and facilitating immediate spoken word retrieval in our patients. Secondly, we predicted that different cue-types would have differential effects on naming performance (considering that they convey different amounts of information about the to-be-retrieved word), and would rely on differential activation within the bilateral residual speech network supporting spoken word retrieval in our brain damaged patients.

## Materials and methods

### Patients

**Figure 1:**
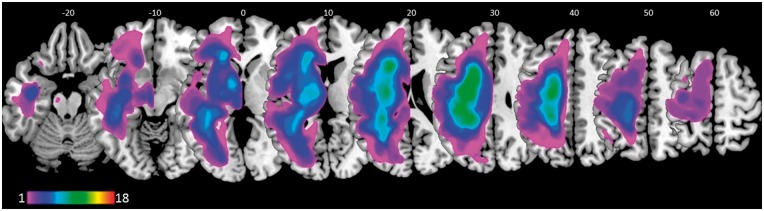
**Lesions overlap in our sample of patients.** Colour range indicates the amount of overlap expressed as number of patients (colour bar). Numbers on the top represent *z* MNI coordinates of brain sections, displayed in neurological convention (i.e. L is L, R is R).

Eighteen right-handed native English speakers with acquired aphasia following a single left-hemisphere stroke participated in the study (see Fig. 1 for a lesion overlap map, Table 1 for demographic and clinical data, and Supplementary Table 1 for a description of lesion locations). All had normal hearing and no previous history of neurological or psychiatric disease, as well as no contraindications to MRI scanning. Inclusion criteria were: (i) anomia as determined by the Boston Naming Test (Kaplan *et al.*, 1983; cut-off <56); (ii) good single word comprehension as assessed by the spoken words comprehension subtest of the Comprehensive Aphasia Test (Swinburn *et al.*, 2005); (iii) relatively spared ability to repeat single monosyllabic words and non-words from the Psycholinguistic Assessments of Language Processing in Aphasia (Kay *et al.*, 1992); (iv) absence of speech apraxia as determined by the Apraxia Battery for Adults (Dabul, 2000); and (v) spared or partially spared left inferior frontal cortex. All gave written informed consent to take part in the study, which was approved by the Central London Research Ethics Committee, and conducted in accordance with the ethical principles stated by the Declaration of Helsinki.

**Table 1:** Demographic and clinical data of the patients

Patient ID	Sex	Age	Lesion volume (cm^3^)	Months post-stroke	BNT	CAT	PALPA 9	PALPA 8	Hours of training
P1	M	64	171	78	47	15	20	6	40
P2	F	49	44	17	12	15	21	6	31
P3	M	54	294	78	14	11	10	0	77
P4	M	41	234	65	28	14	24	8	116
P5	M	49	144	57	34	15	17	2	50
P6	M	66	109	61	52	15	24	6	63
P7	F	44	82	72	34	14	24	10	59
P8	M	54	95	34	35	15	24	8	70
P9	M	67	341	47	42	14	24	9	85
P10	M	41	75	8	23	13	23	8	89
P11	M	63	139	264	51	15	24	9	81
P12	M	47	314	52	16	15	22	6	77
P13	M	56	150	40	1	14	18	2	61
P14	F	60	104	121	27	13	22	7	120
P15	M	41	114	18	42	14	21	3	43
P16	F	21	155	33	18	15	20	3	108
P17	F	47	161	53	9	9^a^	12	0	76
P18	F	43	165	5	21	15	23	1	67
**Mean (SD)**		**50 (12)**	**161 (84)**	**61 (58)**	**28 (15)**	**14 (2)**	**21 (4)**	**5 (3)**	**73 (25)**
		**Max score possible**			**60**	**15**	**24**	**10**	

BNT = Boston Naming Test; CAT = Comprehensive Aphasia Test (spoken words comprehension sub-test); PALPA = Psycholinguistic Assessments of Language Processing in Aphasia (PALPA 9 = monosyllabic words repetition, PALPA 8 = monosyllabic non-words repetition). ^a^Although not normal, P17’s speech comprehension abilities were above chance, and all errors were semantic in nature (e.g. apple for pear).

### Stimuli

In Experiment 1, stimuli consisted of 299 black and white line drawings of objects adapted from the International Picture-Naming Project (Szekely *et al.*, 2004; http://crl.ucsd.edu/experiments/ipnp/index.html). All object names were monosyllabic, consonant-vowel-consonant in terms of phonological structure, and had high name agreement (i.e. at least 75% of test subjects produced the same target name). Monosyllabic words were used to minimize any effect of articulatory challenge.

In Experiment 2, a subset of 107 stimuli out of the 299 from Experiment 1 was used. In order to increase patients’ naming accuracy (and hence optimize the functional MRI design efficiency), this subset of stimuli was chosen by selecting items with the highest frequency ratings. Each picture was presented simultaneously with an auditory cue. Auditory cues were either: (i) a whole word cue (e.g. for the picture of a car, /ka:r/); (ii) an initial phoneme segment (e.g. /ka/); (iii) a final phoneme segment (/a:r/); or (iv) an unintelligible spectrally rotated noise-vocoded auditory control cue (noise), a condition successfully used in previous functional MRI studies (Scott *et al.*, 2000; Narain *et al.*, 2003; Obleser *et al.*, 2007) See Supplementary material for full details on how the auditory cues were generated.

### Anomia treatment

Patients were presented with 150 monosyllabic high frequency to-be-treated items taken from the pool of 299 used in the free-naming experiment (Experiment 1, previous section; 149 items were therefore untreated). Fifty-four items out of 150 were predetermined to be treated in all patients and also used as picture naming stimuli in the functional MRI scanner; whereas 53 of the untreated 149 items were used as naming stimuli in the scanner (Experiment 2). The remaining 96 to-be-treated items (150–54) and 96 untreated items (149–53) for each patient were determined on the basis of their individual pretreatment naming performance (in terms of accuracy) in Experiment 1. This ensured that each patient’s naming performance for the to-be-treated (96 + 54 = 150) and untreated (96 + 53 = 149) word lists were matched at baseline (i.e. that no bias occurred by chance between the two pools of items). These subject-specific items were not used in the functional MRI experiment (Experiment 2). Patients were given a laptop and asked to complete a minimum of 2 h of naming practice daily over a 6-week period. The pictures and auditory cues were presented using the ‘StepByStep’ aphasia treatment software (http://www.aphasia-software.com). The naming practice was designed to be completed in an error-reducing manner (Fillingham *et al.*, 2003, 2006). For example, in naming a picture of a car the patient was asked to name it three times: (i) after a whole word auditory cue /ka:r/; (ii) after an initial phonemic cue /ka/; (iii) after a whole word cue again. Only then would the patient proceed to the next item to be named. Patients completed on average a total of 73 h of naming practice (Table 1). This is within one standard deviation (SD) of the mean total dose of treatment showing a positive impact on aphasic patients’ communicative ability, as found by Bhogal *et al.* (2003) in their meta-analysis of aphasia treatment studies.

### Procedure

**Figure 2:**
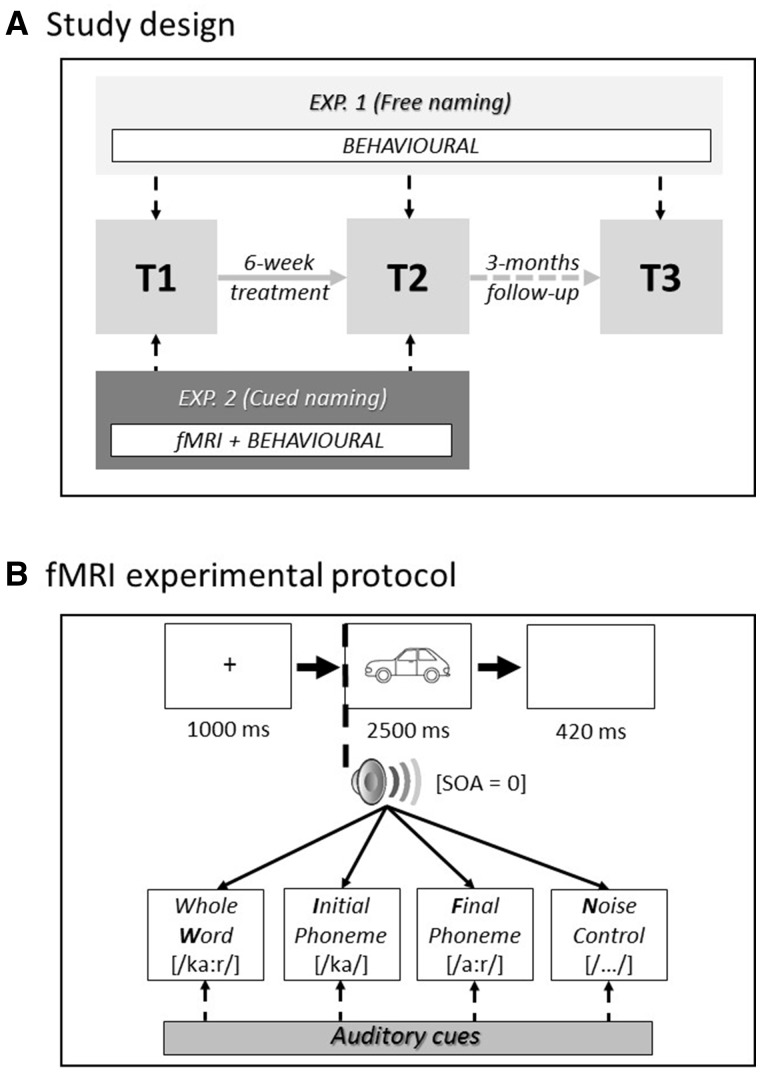
**Study design (A) and functional MRI experimental protocol (B).** fMRI = functional MRI; SOA = stimulus-onset asynchrony.

Experiments 1 and 2 were run in separate sessions, no more than 2 days apart at each of the testing time points (see Fig. 2 for details of study design and functional MRI experimental protocol, *cf*. Supplementary material). In both experiments, patients performed a picture naming task and were instructed to name each picture as quickly and as accurately as possible. Recordings of spoken responses were reviewed offline to score naming accuracy and determine trial-specific reaction time (RT) for each patient. We scored naming accuracy consistent with the standardized Comprehensive Aphasia Test guidelines (Swinburn *et al.*, 2005): verbal, phonemic, neologistic, and dyspraxic errors were not accepted; dysarthric distortions were permitted provided it was clear that each phoneme within the word had been correctly selected.

In Experiment 1, patients were asked to name 299 pictures without the aid of any auditory cue before (T1) and after (T2) the 6-week anomia treatment programme, plus at follow-up 3 months later (T3). In Experiment 2, patients performed an auditory-cued picture naming task in the scanner, at two time points (T1 and T2). Four functional runs were acquired within each scanning session at both time points. Each of the 107 picture stimuli was presented once within each functional run accompanied simultaneously (stimulus-onset-asynchrony = 0 ms) (Supplementary material) with one of four different cue-types (whole word, initial phoneme, final phoneme or noise-control). The order of pictures and accompanying cues was pseudo-randomized (i.e. avoiding more than three trials with the same cue-type), and the order of presentation was counterbalanced both within and across patients.

### Behavioural analyses

**Figure 3:**
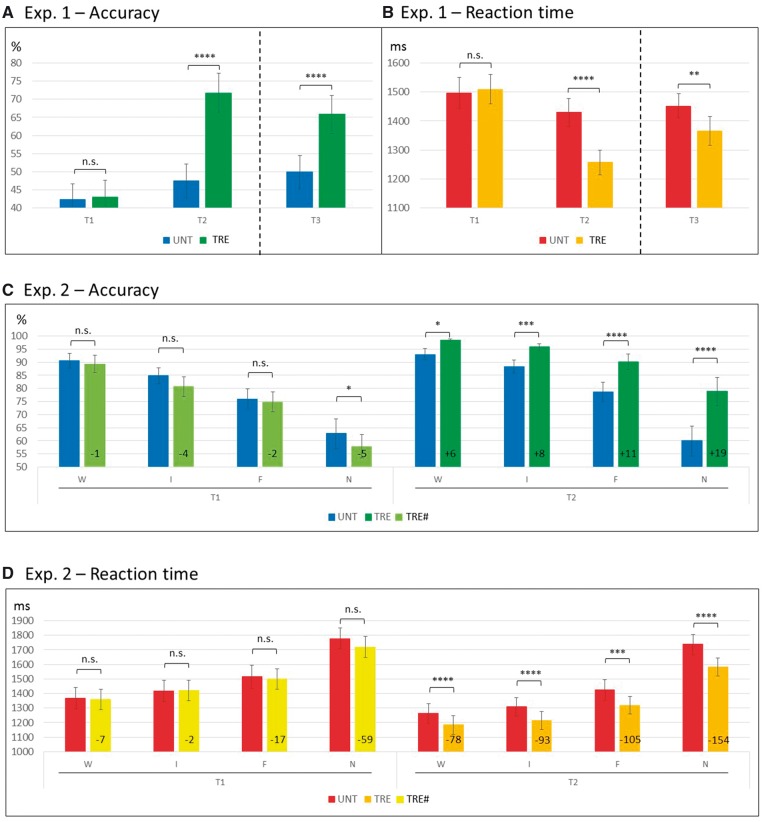
**Behavioural results of Experiment 1 (A–B) and Experiment 2 (C–D).** Dispersions represent standard errors of the mean (SEM). Significance of *post hoc* comparisons: *P ≤ 0.05; **P ≤ 0.01; ***P ≤ 0.005; ****P ≤ 0.001; n.s. = non-significant. For Experiment 1, ANOVAs reported are run across T1 and T2 only, for consistency with all other analyses (results at T3 are reported to show performance at follow-up). Note that (**C**) illustrates that the differences between untreated and ‘to-be-treated’ items at T1 were either non-significant or counter the predicted direction (i.e. UNT > TRE#), whereas at T2 all differences were significant and in line with the predicted direction (TRE > UNT). % = percentage of correct responses; F = final; I = initial; N = noise; TRE = treated items (at T1, TRE# = ‘to-be-treated' items); UNT = untreated items; W = word.

To test the statistical significance of anomia treatment in Experiment 1 we conducted two repeated measures 2 × 2 ANOVAs (one on naming accuracy and one on RT) (Table 2 and Fig. 3A and B), with Time (T1, T2) and Treatment (treated items, untreated items) as within-subject variables. At T1, all items were untreated, but we kept them conceptually separated to check for any potential bias between the stimuli pools (Supplementary material). Hence, treated items at T1 are actually the about ‘to-be-treated’ items. We predicted a significant Time × Treatment interaction. The size of anomia treatment effects directly after treatment (T2 versus T1) and maintenance 3 months later (T3 versus T1) were quantified using both unstandardized and standardized (i.e. Cohen’s *d*) effect sizes (see Supplementary material for calculation).

**Table 2:** Results of behavioural analyses in Experiment 1 and Experiment 2 (ANOVAs)

EXPERIMENT 1				
	Accuracy			
	F	DF-b	DF-w	*P*
**Time**	65.427	1	17	<0.001
**Treatment**	69.163	1	17	<0.001
**Time × Treatment**	29.109	1	17	<0.001

	**Reaction time**			
	**F**	**DF-b**	**DF-w**	***P***

**Time**	11.766	1	17	<0.001
**Treatment**	29.170	1	17	<0.001
**Time × Treatment**	23.859	1	17	<0.001

**EXPERIMENT 2**				
	**Accuracy**			
	**F**	**DF-b**	**DF-w**	***P***

**Time**	14.377	1	17	0.001
**Treatment**	10.672	1	17	0.005
**Cueing**	29.050	3	51	<0.001
**Time × Treatment**	24.279	1	17	<0.001
**Time × Cueing**	1.266	3	51	0.296
**Treatment × Cueing**	3.701	3	51	0.017
**Time × Treatment × Cueing**	11.145	3	51	<0.001

	**Reaction time**			
	**F**	**DF-b**	**DF-w**	***P***

**Time**	12.275	1	17	0.003
**Treatment**	26.498	1	17	<0.001
**Cueing**	61.631	3	51	<0.001
**Time × Treatment**	17.216	1	17	0.001
**Time × Cueing**	3.170	3	51	0.032
**Treatment × Cueing**	3.998	3	51	0.012
**Time × Treatment × Cueing**	0.145	3	51	0.932

*F* = *F*-test; DF-b = degrees of freedom between; DF-w = degrees of freedom within; *P* = *P*-values.

In Experiment 2, two repeated measures 2 × 2 × 4 ANOVAs were conducted (one on accuracy and one on correct RT) (Table 2 and Fig. 3C and D), with Time (T1, T2), Treatment (treated, untreated) and Cueing (whole word, initial phoneme, final phoneme, noise-control) as within-subject variables. Again, treated items at T1 are actually the about ‘to-be-treated’ items. Here, we focussed on both the effectiveness of the anomia treatment and the effect of cue-types, predicting significant interactions between Time and Treatment, and between Treatment and Cueing. Significance threshold for reported results was set to *P* < 0.05 throughout.

### Imaging acquisition and analysis

Whole-brain imaging was performed on a 3 T Siemens TIM-Trio system (Siemens) at the Wellcome Trust Centre for Neuroimaging. T_2_*-weighted echo-planar images with BOLD contrast were acquired using a 12-channel head coil. Each image comprised 48 AC/PC-aligned axial slices with sequential ascending acquisition, slice thickness = 2 mm, inter-slice gap = 1 mm, in-plane resolution = 3 × 3 mm. Volumes were acquired with a repetition time = 3360 ms, and the first six volumes of each session were discarded to allow for T1 equilibrium effects. At each time point (T1 and T2), a total of 180 volume images (174 volumes of interest and six dummy scans) were acquired in four consecutive runs, each lasting ∼10 min. Prior to the first functional run of each scanning session, a gradient field map was acquired for each patient for later B0 field distortion correction of functional images. The same scanner and hardware were used for the acquisition of all images.

Functional data were preprocessed (see Supplementary material for details) and analysed using Statistical Parametric Mapping software (SPM12; www.fil.ion.ucl.ac.uk/spm) running under Matlab 2015a (MathWorks, Natick, MA). Statistical analyses were first performed in a subject-specific fashion. Nine conditions per each time point (i.e. four cue-types × two treatment levels, plus incorrect responses) were modelled separately as events convolved with the SPM canonical haemodynamic response function. We used the presentation of the concurrent picture and auditory cue as the onset of the event to model the preparatory naming response. Movement realignment parameters were included as covariates of no interest. The resulting stimulus-specific parameter estimates were calculated for all brain voxels using the General Linear Model. At the second level, 16 conditions of interest were modelled (four cue-types × two treatment levels × two time points, discarding incorrect responses, and merging sessions across T1 and T2), modelling subjects as a random factor. Significance threshold was set to *P* < 0.05 (FWE-corrected for multiple comparisons across the whole brain or within a region of interest, see below). Anatomical labelling was determined by using the Automated Anatomical Labeling atlas (Tzourio-Mazoyer *et al.*, 2002).

## Results

### Experiment 1: Free-naming

#### Accuracy

Results showed a significant Time × Treatment interaction (Table 2, Fig. 3A and Supplementary Table 2). As predicted, naming of treated items was more accurate at T2 (72%) than T1 (43%), and this unstandardized effect size (29%) was significantly greater (*P* < 0.001) than the difference (5%) between untreated items at T2 (47%) and T1 (42%; *P* = 0.006). There were also significant main effects of Time and Treatment (both driven by improvements for treated items at T2). Treatment effects remained significant at T3 (Fig. 3A), indicating that naming gains were maintained 3 months later. Cohen’s *d*-values indicated large anomia treatment effect sizes: 3.45 for the immediate post-treatment effects (comparison T2 versus T1), and 1.83 for longer-term naming changes (T3 versus T1).

#### Reaction time

Results here mirrored the accuracy results, with a significant Time × Treatment interaction (Table 2 and Fig. 3B). Naming of treated items was faster at T2 (1257 ms) than T1 (1510 ms), and this unstandardized effect size (17%) was significantly greater (*P* < 0.001) than the difference (4%) between untreated items at T2 (1430 ms) than T1 (1496 ms; *P* = 0.206). Again, there was a significant main effect of both Time (faster at T2 than T1) and Treatment (driven by the treatment effect at T2). Here Cohen’s *d*-values were smaller than the effects on naming accuracy (−0.88, i.e. reduced RT post-treatment for the comparison T2 versus T1; and −0.45 for T3 versus T1).

In summary, our anomia treatment approach using phonemic cues resulted in significant, effective and long-lasting (i.e. maintained 3 months later) improvements in patients’ naming accuracy and efficiency (RT) that was greater for treated items. There was no evidence to support a naming accuracy versus speed trade-off in treatment gains. Importantly, none of the indices of treatment outcome (i.e. differences between treated items at different time points) correlated with variables such as age, months post-stroke, hours of training, and lesion volume (Supplementary Table 3).

### Experiment 2: Cued-naming behavioural data

#### Accuracy

As predicted—and consistent with Experiment 1—the Time × Treatment interaction (see Table 2 and Fig. 3C for ANOVAs results) showed that naming of treated items was more accurate at T2 (91%) than T1 (76%), and this unstandardized effect size (15%) was significantly greater (*P* < 0.001) than the difference between untreated items at T2 (80%) and T1 (79%). There were also significant main effects of Time (more accurate at T2 than T1) and Treatment (driven by the treatment effects at T2).

There was also a significant main effect of Cueing. Across time points, accuracy was highest for word cues (93%) and least for no cues (65%). Time × Treatment × Cueing interaction showed that cueing modulated the effect of treatment on naming accuracy (i.e. treated versus untreated items) at T2 compared to T1. This also resulted in a significant Treatment × Cueing interaction, whereas the Time × Cueing interaction was not significant.

Having factored out any potential bias between the two pools of naming stimuli (‘to-be-treated’ versus untreated items) at T1 (*cf*. Fig. 3C and Supplementary material), we focus on *post hoc* analyses of naming differences at T2. Naming for treated (relative to untreated) items improved as follows for each cue-type: whole words from 93% to 99% (+6%), initial phonemes from 88% to 96% (+8%), final phonemes from 79% to 90% (+11%), noise-control from 60% to 79% (+19%; all *P* < 0.05). Note the probable ceiling effects observed here where at T2 the degree of naming improvement post-treatment across cue-types was inversely proportional to the degree of possible improvement from T1 scores (i.e. the more room there was for improvement, the more they improved with treatment).

#### Reaction time

These results mirrored the naming accuracy results with one exception. Here, the Time × Treatment × Cueing interaction was not significant. The Time × Treatment interaction (Table 2 and Fig. 3D) showed that naming of treated items was faster at T2 (1326 ms) than T1 (1499 ms), and this unstandardized effect size (12%) was significantly greater (*P* < 0.001) than the difference between untreated items at T2 (1433 ms) and T1 (1519 ms). The Time × Cueing interaction showed that RT was modulated differently by cue-types at T1 and T2. The Treatment × Cueing interaction showed that reductions in RT following treatment were significantly modulated by cue-types.

Again, having factored out any potential bias between the two pools of stimuli (‘to-be-treated’ versus untreated items) at T1 (*cf*. Fig. 3D and Supplementary material), we focus on *post hoc* analyses of RT differences at T2. At T2 naming RT for treated (relative to untreated) items improved as follows for each cue-type: whole words 78 ms (+6%), initial phonemes 93 ms (+7%), final phoneme 105 ms (+7%), noise-control 154 ms (+9%; all *P* < 0.005). Consistent with the improvements observed in naming accuracy post-treatment, the naming gains in RT were visible for all treated items across all cue-types, and cueing effects were observed irrespective of whether they were used in treatment or not. Results also showed significant main effects of Time (T2 faster than T1), Treatment (at T2), and Cueing (fastest for word cues and slowest for no cues).

Overall, our behavioural results from Experiment 2 on a cued-naming task replicated those from Experiment 1 on free-naming task. There was a significant positive effect of the anomia treatment on the patients’ naming performance, improving both accuracy and RT for treated items more than untreated items. Furthermore, Experiment 2 showed that the effect of treatment was greatest in the noise-control naming trials. These cues had no priming effects on naming performance, so data from these trials are most similar to free-naming (i.e. non-facilitated) performance (Experiment 1).

### Neuroimaging data

#### Time

First, we assessed whether the simple effect of time elapsed between the two measures (T1, T2) had any impact on brain activity. Both contrasts T1 > T2 and T2 > T1 did not show any significant results even though accuracy was higher and RT was faster at T2.

#### Immediate facilitation of naming (cueing)

**Figure 4:**
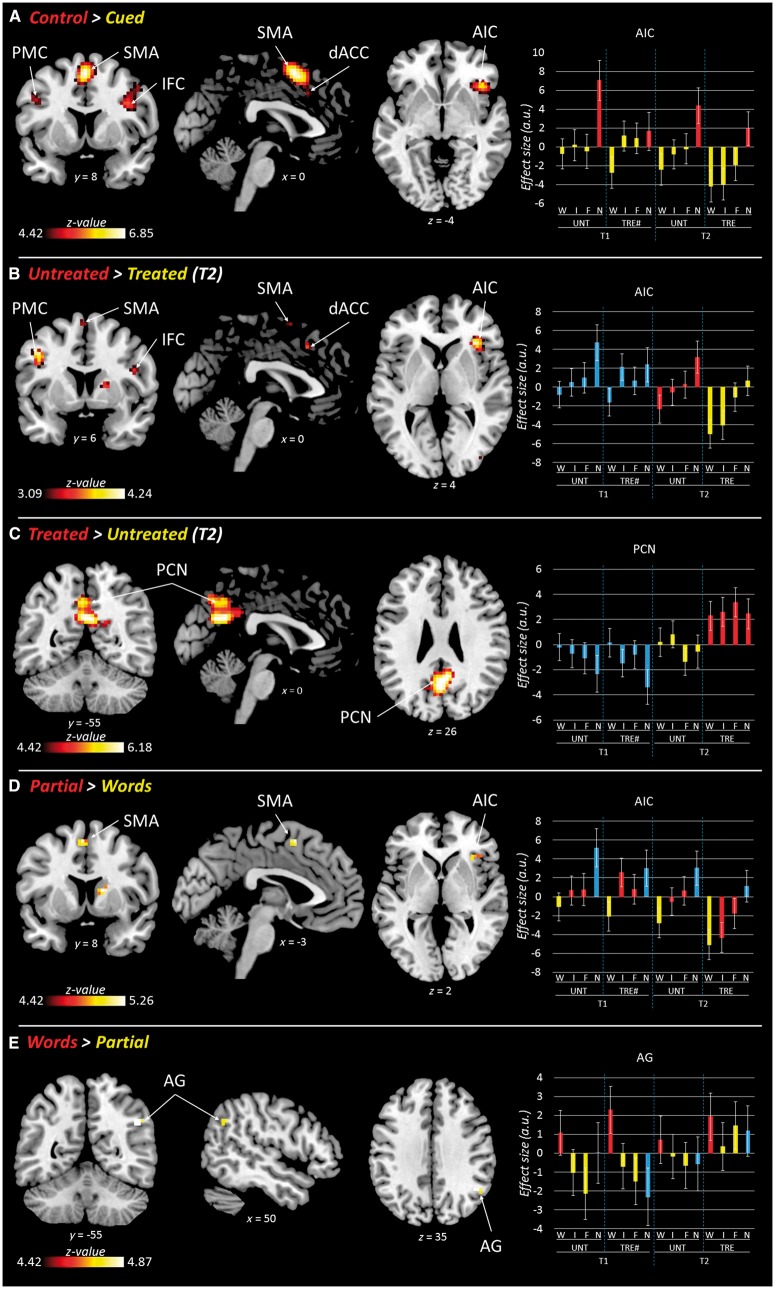
**Functional MRI results.** (**A**) Reductions in BOLD response related to immediate facilitation of naming. (**B**) Reductions in BOLD response related to long-term facilitation of naming. (**C**) Increased BOLD response for treated (as compared to untreated) items. (**D**) Activations related to partial (i.e. initial and final phonemes) cues processing. (**E**) Activations related to whole word cues processing. Results are displayed at *P* < 0.05 (FWE-corr.), except in (**B**), where they are displayed at *P* < 0.001 (unc.) for cluster extent, without correction at cluster-level (to allow for small-volume correction). Red and yellow bars refer to the conditions compared (red > yellow, as in the title of each contrast). Blue bars are conditions not included in the contrasts. a.u. = arbitrary unit; AG = angular gyrus; AIC = anterior insular cortex; Control = control trials (i.e. noise); Cued = cued trials (i.e. word, initial, and final); dACC = dorsal anterior cingulate cortex; F = final; I = initial; IFC = inferior frontal cortex; N = noise; Partial = partial cues (i.e. initial and final phonemes); PCN = precuneus; PMC = premotor cortex; SMA = supplementary motor area; TRE = treated items (TRE# = ‘to-be-treated' items); UNT = untreated items; W = word; Words = whole word cues; *x y z* = MNI coordinates of brain sections. Sections are displayed in neurological convention.

Second, we identified the neural network associated with the immediate facilitation effects of phonemic cues paired during naming by contrasting items paired with (i) whole word, initial and final phoneme cues (‘Cued’); and (ii) noise-control cue (‘Control’). The contrast Control > Cued resulted in a significant neural priming effect, i.e. reduced BOLD response for cued items as compared to noise-control items. Bilateral reductions were observed in the dorsal anterior cingulate cortex, supplementary motor area, premotor cortex (precentral gyri), and opercular inferior frontal cortex. Right lateralized reductions were observed in the anterior insula, extending into the adjacent orbital and triangular inferior frontal cortex, plus in the posterior superior temporal sulcus extending into the adjacent superior and middle temporal cortices (Table 3 and Fig. 4A). These results defined a functional ‘cueing network’ supporting naming performance. The reverse contrast (Cued > Control) did not show any significant result, although a sub-threshold peak was identified in the precuneus (Table 3).

**Table 3:** Functional MRI results

Contrast	Control > Cued				Untreated > Treated			
Region	*x y z*	*P*(FWE)	K	Z	*x y z*	*P*(SVC)	K	Z
R anterior insular cortex	42 23 −4	<0.001	112	6.22	33 26 5	0.003	108	4.17
R/L supplementary motor area	0 8 56	<0.001	271	6.85	−3 5 65	0.082	163	3.25
R dorsal anterior cingulate cortex	9 20 35			5.74	3 20 44	0.022		3.67
L dorsal anterior cingulate cortex	−6 20 38			5.55	−6 17 35	0.944	1	1.68
R inferior frontal gyrus (opercular)	42 11 29	<0.001	109	6.12	45 8 23	0.022	87	3.66
R precentral gyrus	48 8 44			5.02	−	−	−	−
L inferior frontal gyrus (opercular)	−42 5 29	<0.001	25	5.14	−	−	−	−
L precentral gyrus	−45 5 38			4.92	−45 5 38	0.007	25	3.98
R middle temporal cortex	60 −46 11	0.003	10	5.01	−	−	−	−

**Contrast**	**Cued > Control**				**Treated > Untreated**			
**Region**	***x y z***	***P*(unc.)**	**K**	**Z**	***x y z***	***P*(FWE)**	**K**	**Z**

R precuneus	6 −55 29	0.003	4	2.70	6 −55 26	<0.001	349	6.18
L precuneus	−	−	−	−	−3 −58 41			5.78

**Contrast**	**Partial > Words**				**Initial > Final**			
**Region**	***x y z***	***P*(FWE)**	**K**	**Z**	***x y z***	***P*(unc.)**	**K**	**Z**

R anterior insular cortex	33 26 2	0.004	9	4.93	−	−	−	−
R inferior frontal gyrus (triangular)	42 26 2			4.71	−	−	−	−
L supplementary motor area	−3 8 56	0.003	11	5.06	−	−	−	−
R caudate/putamen	15 5 11	<0.001	25	5.26	−	−	−	−

**Contrast**	**Words > Partial**				**Final > Initial**			
**Region**	***x y z***	***P*(FWE)**	**K**	**Z**	***x y z***	***P*(unc.)**	**K**	**Z**

R angular gyrus	45 −52 32	0.004	9	4.87	−	−	−	−

**Contrast**	**Final > Words**				**Initial > Words**			
**Region**	***x y z***	***P*(FWE)**	**K**	**Z**	***x y z***	***P*(unc.)**	**K**	**Z**

R anterior insula	33 23 2	0.012	4	4.68	33 26 2	0.001	225	3.84
R inferior frontal cortex (triangular)	48 26 17	0.020	2	4.77	45 26 5			3.36
L supplementary motor area	−3 8 56	<0.001	30	5.53	−3 8 56	0.001	4	3.20
R caudate/putamen	15 5 11	<0.001	29	5.46	15 5 11	0.001	225	3.66

**Contrast**	**Words > Final**				**Words > Initial**			
**Region**	***x y z***	***P*(FWE)**	**K**	**Z**	***x y z***	***P*(unc.)**	**K**	**Z**

R angular gyrus	45 −55 32	0.028	1	4.73	51 −55 35	0.001	96	4.01

R = right; L = left; *x y z* = MNI coordinates; K = cluster size; Z = z-scores; FWE = family-wise error corrected *P*-values; SVC = small-volume corrected *P*-values within the volume of interest resulting from the contrast ‘Control > Cued'; unc. = uncorrected *P*-values (reported for completeness).

#### Long-term facilitation of naming (treatment)

Third, we characterized the long-term facilitation effects of anomia treatment on naming (i.e. effect of treated versus untreated items at T2 only). The contrast Untreated > Treated did not show any significant activations at the whole-brain level. When the statistical threshold was lowered (*P* < 0.001 unc.) and a small-volume correction applied within the ‘cueing network’ identified with the orthogonal contrast Control > Cued, the following regions were identified: right anterior insula, dorsal anterior cingulate cortex, and opercular inferior frontal cortex; plus left premotor cortex (Table 3 and Fig. 4B). The effect of treatment in these areas mirrored that seen for cueing—i.e. a reduction in BOLD response when naming response was facilitated by treatment (or cueing). The reverse contrast Treated > Untreated showed a significant cluster of activation located in the precuneus and the adjacent posterior cingulate cortex (Fig. 4C), but no significant activation within the ‘cueing network’.

#### Partial cues versus whole-word cues (within-cues)

Fourth, we investigated whether the different auditory cue-types that contained varying amounts of phonemic/semantic information—i.e. whole words, initial phonemes, final phonemes—had a differential effect on brain activity during picture naming, considering that differential behavioural effects of cue-types were identified in terms of naming accuracy and RT between them (Fig. 3C and D). The contrasts Initial > Final and Final > Initial did not show any significant results, so we grouped Initial and Final cues together (i.e. ‘Partial’) for further analyses. The contrast Partial > Words identified significant clusters of activation in the left supplementary motor area, and right anterior insula and triangular inferior frontal cortex (within the ‘cueing network’), as well as in the right basal ganglia (Table 3 and Fig. 4D). The contrast Words > Partial showed a significant activation in the right angular gyrus (Table 3 and Fig. 4E). Importantly, results were replicated when initial and final cues were compared with words separately (i.e. Initial > Words and Final > Words, and vice-versa; *cf*. Table 3).

In summary, neuroimaging results showed a substantial overlap between the neural mechanisms implicated in immediate and long-term facilitation of picture naming in chronic aphasic stroke patients. Furthermore, different auditory cues facilitating picture naming recruited different hubs within the residual naming network with whole words activating right angular gyrus, and partial cues bilateral frontal regions.

#### Relationship between naming performance and brain activity

As a final step, we investigated whether and how individual patients’ change in brain activity (BOLD response: Experiment 2) following anomia treatment was related to their change in free-naming performance (Experiment 1). More specifically, we tested—for successfully named items only—whether the change in BOLD response for noise-cued pictures (Experiment 2) correlated with a change in un-cued naming RT (speed; Experiment 1). The noise-control cues had no priming effects on naming performance so data from these trials are most similar to free-naming (i.e. non-facilitated) performance (Experiment 1). First, we extracted the adjusted BOLD response values (noise-control condition only) on an individual basis from each of the peak regions identified with the contrast Untreated > Treated shown to be sensitive to anomia treatment (right anterior insula, inferior frontal cortex and dorsal anterior cingulate cortex, plus left premotor cortex) (Table 3). Then, we computed correlations between individual differences in BOLD response for treated and untreated items at T2 (T2_UNT-TRE) and the corresponding differences between naming RT scores (in Experiment 1).

**Figure 5:**
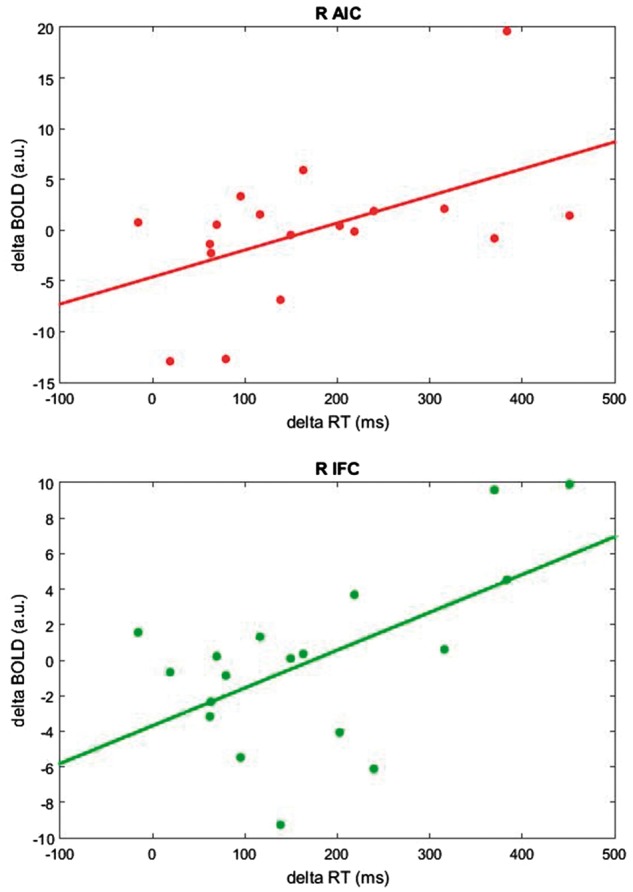
**Correlation between behaviour and brain activity in the right anterior insular cortex (A) and in the right inferior frontal cortex (B).** Plots show the relationship between naming efficiency (computed as the difference between mean RT: untreated-treated items at T2) and corresponding changes in BOLD response (i.e. untreated-treated) extracted during the noise-control condition. A greater improvement in naming efficiency (delta RT) is associated with greater changes in BOLD response (delta BOLD). R AIC = right anterior insular cortex; R IFC = right inferior frontal cortex; a.u. = arbitrary unit.

Patients’ difference in BOLD response following treatment significantly correlated with treatment-induced improvements in naming efficiency in the right anterior insula (r = 0.51, *P* = 0.031) and right inferior frontal cortex (r = 0.57, *P* = 0.013), but not in the right dorsal anterior cingulate cortex and left premotor cortex. These correlations were positive: patients with the greatest right frontal decreases in BOLD response following treatment also had the greatest improvement in naming RT (biggest change in naming speed, i.e. faster) (Fig. 5).

## Discussion

In this study we aimed to understand, for the first time, how speech production in aphasic patients is supported by neural mechanisms both during anomia treatment (i.e. the therapeutic process itself), and following anomia treatment (i.e. longer-term speech outcome). To address this, we delivered a high-dose, cued-naming anomia treatment programme to a group of chronic aphasic stroke patients (Experiment 1) and—in the same patients—utilized a functional MRI cued-naming paradigm that mirrored the therapy approach, before and directly after the treatment (Experiment 2). We found significant treatment-specific effects, both in terms of naming improvements (RT and accuracy) and brain activity (BOLD signal), with immediate facilitation of naming performance and longer-term facilitation of naming (post-treatment) supported by the same bilateral residual neural network. Furthermore, patients’ treatment outcome (free-naming efficiency – RT) was directly related to neural priming (decreases in BOLD signal) in right frontal cortices. These data suggest that language homologue regions in the right hemisphere play an active and facilitatory role in chronic aphasic stroke patients’ anomia treatment response. Interestingly, naming accompanied by whole word cues activated the right angular gyrus, while partial cues (initial and final phonemic cues) activated bilateral frontal regions.

Like our first experiment (Experiment 1), there have been many behavioural studies focused on changes in aphasic patients’ speech performance (naming) following anomia treatment using a cued-naming approach, i.e. treatment outcome changes (Raymer *et al.*, 2007; Kendall *et al.*, 2008; Lambon Ralph *et al.*, 2010; Best *et al.*, 2013). This approach to anomia treatment works, even in the chronic stage post-stroke, and when given a high dose patients can make significant long-lasting speech gains (Bhogal *et al.*, 2003; Brady *et al.*, 2016; also *cf*. Breitenstein *et al.*, 2017). However, treatment effects are item-specific with little-to-no generalization of improvements when naming untrained items (i.e. only naming of treated items improves). Consistent with this, we found significant treatment effects for treated items only, with long-lasting maintenance of naming gains observed 3 months post-treatment. The size of our treatment effects was large and arguably greater and/or longer-lasting than those reported in previous neuroimaging studies (*cf*. Fridriksson *et al.*, 2007; Vitali *et al.*, 2007; Rochon *et al.*, 2010; Abel *et al.*, 2014; van Hees *et al.*, 2014; Dignam *et al.*, 2016). This is likely due to the higher dose of treatment we delivered (73 h on average) and the larger pool of items we treated (*n* = 150).

Surprisingly, none of these studies have investigated how their adopted anomia treatments work at a brain systems level and lead to the observed speech change, i.e. brain plasticity underlying word retrieval during—and as a longer-term consequence of—therapy. In our study we intended to directly address this question. To do this, we used a functional MRI task (Experiment 2) based on the same approach as used in our behavioural therapy (Experiment 1). This enabled us to identify brain regions sensitive to our anomia treatment approach both immediately (during) and directly after (T2) the intervention. We found that immediate and long-term naming facilitation relied on a common bilateral neural network. This included in the right hemisphere the anterior insula, inferior frontal cortex, and dorsal anterior cingulate cortex; and in the perilesional left hemisphere the premotor cortex and supplementary motor area.

That the right hemisphere was consistently recruited during successful picture naming both during and directly post-treatment in our chronic aphasic patients is striking. There has long been a debate in the literature about the role of the right hemisphere in speech and language recovery. While some authors have claimed that its involvement is detrimental (Naeser *et al.*, 2005; Winhuisen *et al.*, 2005), others have argued that it might be beneficial (Blasi *et al.*, 2002; Crinion and Price, 2005). More recently, it has been shown that language production outcome in chronic aphasic patients is associated with structural changes in language homologue areas in the right hemisphere (Wan *et al.*, 2014; Pani *et al.*, 2016; Xing *et al.*, 2016).

The primary supporting evidence of an inhibitory role of the right hemisphere in aphasic patients’ spoken language function comes from neurostimulation studies. Here, low frequency repetitive (i.e. inhibitory) transcranial magnetic stimulation applied to the right inferior frontal cortex (Broca’s area homologue) has been associated with improved naming abilities (Martin *et al.*, 2004; Naeser *et al.*, 2005; for a meta-analysis, see also Ren *et al.*, 2014). While the contradictions in the literature about this still need to be solved, it has been suggested that the mechanisms elicited by neurostimulation might not be as straightforward as previously outlined, inviting us to interpret this with caution. Indeed, issues such as the nature of the relationship between inhibitory and excitatory balance within a reorganizing bi-hemispheric language network, the relationship between short- and long-term effects of neurostimulation, and the interplay between specific subparts of the language network and their right hemisphere homologues are still rather unclear (Turkeltaub, 2015).

The set of prefrontal areas we identified included not only regions that might be considered right homologues of well-known speech and language network hubs (inferior frontal cortex, anterior insula, premotor cortex—especially related to phonological processing) (Bamiou *et al.*, 2003; Liakakis *et al.*, 2011), but also regions involved in executive processes and domain-general or multiple-demand systems (dorsal anterior cingulate cortex and supplementary motor area) (Duncan, 2010; Fedorenko *et al.*, 2013; Fedorenko, 2014; Hertrich *et al.*, 2016). Within our study design, we cannot tease apart the different role each of these regions contributed to naming. However, it is interesting to note that these regions were consistently modulated by task difficulty, i.e. hard versus easy naming conditions, as indexed by RT and accuracy. For example, for each of the functional MRI contrasts: Control > Cued, Untreated > Treated, and Partial > Words, BOLD activity within these regions was higher (Table 3).

In contrast, a posterior hub (precuneus) was associated with increased activation for the ‘easier’ naming conditions (Treated > Untreated; Cued > Control). The precuneus is a core hub of the so-called ‘default mode network’ (Raichle *et al.*, 2001; Greicius *et al.*, 2003), whose activity has been shown to be anti-correlated to task engagement (McKiernan *et al.*, 2003; Pfefferbaum *et al.*, 2011). Interestingly, in most previous studies of aphasic patients its activity has been systematically reported to be modulated by behavioural changes following therapeutic interventions (Fridriksson *et al.*, 2007; Vitali *et al.*, 2007; Menke *et al.*, 2009; Fridriksson, 2010; Rochon *et al.*, 2010; Abel *et al.*, 2014, 2015; van Hees *et al.*, 2014), although typically as a secondary or complementary result. Given the sensitivity of this neural structure to task engagement and its possible implication in treatment response, further investigation on the role of the precuneus in language recovery may be of interest in future studies.

Following treatment, only activation change in right anterior insula/inferior frontal cortex correlated significantly with improved naming efficiency (Fig. 5). This indicates a specific involvement of these regions in our patients’ recovery. We interpret this result as a consequence of our anomia treatment: the repeated pairing of cue and picture during treatment primed these right hemisphere regions (decreased BOLD signal) and facilitated more efficient word retrieval (faster and more accurate naming). This treatment approach made a naming task that was hard for the patients at the outset easier by utilizing and optimizing their residual right hemisphere speech networks.

Furthermore, similar neural mechanisms were involved in immediate and long-term facilitation of word retrieval. A significant neural priming effect during naming (i.e. reduced BOLD response) was observed for (i) cued items compared to control items; as well as for (ii) treated items compared to untreated items (i.e. as a consequence of the treatment undertaken). This suggests that neural priming mechanisms within the naming network underlie the patients’ immediate facilitation (faster RT) when naming cued pictures. Treatment (mass practice) then consolidated these mechanisms leading to further neural priming within the same network and faster naming RT when patients named the treated items (T2). This is consistent with cognitive models of speech production proposing that the phonemic cueing approach used in anomia treatment relies upon the same processes underlying cued-picture naming priming effects found in healthy speakers (Best *et al.*, 2002; Nickels, 2002). Phonemic cues presented concurrently with a picture to-be-named prime the retrieval of a correct phonological word form, reduce lexical selection demands, and result in faster naming responses (Miceli *et al.*, 1996).

Indeed, different auditory cues had differential effects on the patients’ immediate naming performance and right hemisphere brain activation patterns. This suggests that they may have been facilitating residual speech functions by tapping into different underlying neural and/or cognitive mechanisms. When given partial cues (initial and final phonemes), picture naming was more demanding/harder (as indexed by slower and less accurate responses) than naming with whole word cues (*cf*. Fig. 4D). Partial cues activated bilateral frontal regions, while whole word cues activated the right angular gyrus (Fig. 4E). Interestingly, this pattern was reversed in the case of treated items, whereby partial cues elicited a BOLD response similar to whole words (*cf*. Fig. 4D and E).

By definition, partial cues share only part of the phonological information of the target word, so that patients still need to retrieve the lexical, phonological and semantic representations of the target word. These cues probably act by priming phonologically similar words, thereby reducing competition among antagonist lexical representations (Aristei *et al.*, 2012; Vitkovitch and Cooper, 2012; Melinger and Abdel Rahman, 2013; Britt *et al.*, 2016; but see Mahon *et al.*, 2007 and Navarrete *et al.*, 2014 for alternative views), eventually improving word search and retrieval in patients. In healthy subjects, the left inferior frontal cortex has been reported to be implicated in phonological processing (Poldrack *et al.*, 1999), word retrieval (Grabowski *et al.*, 1998; Sharp *et al.*, 2005), and articulatory planning (Price, 2010). On the other hand, recent neurostimulation studies have shown that the right inferior frontal cortex is also crucially implicated in phonological processing and object naming (Hartwigsen *et al.*, 2010; Sollmann *et al.*, 2014). In aphasic patients, perilesional areas in the inferior frontal cortex have been shown to play a vital role in aphasia recovery (Fridriksson, 2010; Fridriksson *et al.*, 2012). Moreover, the right homologue of Broca’s area was found activated during word retrieval in patients with lesions to the left inferior frontal cortex (Perani *et al.*, 2003).

In contrast, whole word cues provide the full lexical, phonological and semantic forms of the target word. As such, it could be argued that it is an ‘easier’ naming condition, and indeed patients may not have been using lexical retrieval processes *per se*, but rather word repetition processes to complete the task (Nozari *et al.*, 2010). In healthy subjects, the left angular gyrus has been implicated in semantic processing (Mechelli *et al.*, 2007; Price, 2010; Seghier *et al.*, 2010), language comprehension and sentence processing (Sakai *et al.*, 2001; Dronkers *et al.*, 2004), and verbal working memory (Clark *et al.*, 2001). In aphasic patients, lesions in the left angular gyrus were associated with impaired sentence comprehension and verbal working memory (Newhart *et al.*, 2012), whereas in patients with extensive damage to the language network in the left hemisphere the right angular gyrus was found activated during semantic processing (Sims *et al.*, 2016).

In summary, the present study aimed to understand—for the first time—the neural mechanisms underlying the phonemic cueing therapeutic process involved in anomia treatment, and post-treatment longer-term speech outcome in chronic aphasic patients. Our main result shows that immediate facilitation of naming performance using a cued-naming approach, and long-term facilitation of naming post-treatment are supported by a common bilateral residual neural network, including the anterior insula, inferior frontal cortex, and dorsal anterior cingulate cortex in the right hemisphere; the premotor cortex and supplementary motor area in the left hemisphere.

We have presented a new technique for evaluating the effect of anomia treatment in aphasia patients. The novel protocol we provide can be used as a framework for brain and behavioural plasticity in the damaged brain from which researchers and clinicians can optimize current anomia treatment approaches, develop new treatments, and ultimately improve speech outcome for aphasic patients. Future studies in our lab utilizing this approach will aim to investigate individual patients’ responses and make predictions about the therapeutic outcome. We hope this will lead to better patients’ stratification (e.g. identification of good candidates for this approach), optimizing both treatment path and outcome.

## Funding

D.N. and J.C. are supported by a Wellcome Trust Senior Research Fellowship in Clinical Science (106161/Z/14/Z) awarded to J.C. Data collection was supported by an MRC Clinical Scientist Fellowship (G0701888) awarded to J.C.

## Supplementary material

Supplementary material is available at *Brain* online.

## Supplementary Material

Supplementary Tables

## Abbreviations

BOLD: blood oxygen level-dependent
RT: reaction time
T1: pretreatment measure
T2: post-treatment measure
T3: follow-up measure 3 months after treatment
