# Supplementary material for: Less is more: neural mechanisms underlying anomia treatment in chronic aphasic patients
Source: Brain. 2017 Sep 27;140(11):3039–54. doi: 10.1093/brain/awx234 (PMC5808641; doi:10.1093/brain/awx234)
Supplement: Supplementary Tables [file brain-2017-00663-file010_awx234.pdf]

SUPPLEMENTARY MATERIAL

Stimuli

Generation of noise control cues

The control cues were broadband stimuli derived from the natural single-word speech recordings that were then spectrally rotated and noise-vocoded to remove any phonemic and semantic information. Subjects reported they sounded like words but of a foreign language. To generate the auditory cues, each target name was digitally recorded (at 44.1 kHz) from a male native English speaker in a soundproof room. Word cues were the spoken whole word tokens. To generate the initial and final phoneme cues each whole word token was cropped at either the offset of the vowel to form the initial cue (e.g. /ka/), or the onset of the vowel to form the final cue (/at/). Initial and final cues were then matched for total auditory duration. To generate the control cues, each spoken word cue was spectrally rotated (Blesser, 1972), and then submitted to a noise-vocoding routine (Shannon *et al.*, 1995) using a single level of filter band noise vocoding. This procedure leaves the temporal envelop of the spoken token unaltered and preserves the spectro-temporal complexity, while rendering the auditory signal unintelligible by inverting the frequency spectrum.

Potential bias between pools of stimuli at T1

In order to rule out the presence of any (accidental) potential bias between the two pools of stimuli before the therapy, we computed two-tailed paired t-tests between 'to-be-treated' and 'untreated items' at T1. In Experiment 1, the comparisons showed no significant difference for both accuracy (t=0.45, *P* =0.660) and RT (t=1.00, *P* =0.331). In Experiment 2, the comparisons showed indeed a significant difference for accuracy (t=-2.56, *P* =0.021), but not for RT (t=-1.70, *P* =0.108). Importantly, it should be noticed that the direction of the significant comparison is opposite to our predictions and the effects found at T2 (i.e. TRE>UNT). This means that at T1 'to-be-treated' items were named with a lower accuracy as compared to 'untreated items' (i.e. UNT>TRE).

Procedures

Stimulus-Onset-Asynchrony between pictures and auditory cues

Auditory cues were presented simultaneously with each picture (Stimulus-Onset-Asynchrony – SOA=0ms). The time at which the phonemic cue is delivered has important consequences for priming speech production. Previous studies have found robust phonological priming effects on naming with a SOA of 0ms (Schriefers *et al.*, 1990; Meyer and Schriefers, 1991; Starreveld, 2000; de Zubicaray and McMahon, 2009), whereas priming effects decrease when auditory cues precede visual stimuli (Abel *et al.*, 2009).

functional MRI experimental protocol

Each visual stimulus was displayed for 2500ms, preceded by a 1000ms fixation cross and followed by a blank screen for 420ms. Trials were presented in mini-blocks of six stimuli, separated by fixation-only rest periods of 7840ms in order to optimize the BOLD response (Henson, 2006). The inter-trial interval was set to 3920ms to jitter the onset on each trial across acquired brain volumes and vary the spatial acquisition of the functional MRI data. Overt spoken responses were recorded in the scanner using a dual-channel, noise-cancelling fibre optical microphone system (FOMRI III, http://www.optoacoustics.com). Auditory cues were delivered via an MR-compatible set of headphones (MR Confon, Magdeburg, Germany; www.mr-confon.de).

Analyses

Computation of Cohen's ***d***

The calculation being, mean naming performance change over time for treated items (experimental condition) minus mean performance change over time for untreated items (control condition), (in case of standardized effect size) divided by the standard deviation of performance change over time for untreated items (control condition). For example, the standardized effect size for naming treatment change between T1 and T2 was calculated as follows: [mean(TRE_T2─TRE_T1)]─[mean(UNT_T2─UNT_T1)]/SD(UNT_T2─UNT_T1).

Preprocessing of functional MRI data

All volumes of interest from each patient were realigned and unwarped, using session and subject-specific voxel displacement maps (Hutton *et al.*, 2002). The functional images were then spatially normalized. This step included skull-stripping using BET running under FSL 3.2.0 (http://www.fmrib.ox.ac.uk/fsl), and a subsequent estimation of segmentation parameters using a unified segmentation routine implemented in the Automatic Lesion

Identification (ALI) toolbox (Seghier *et al*., 2008). Finally, functional data were spatially smoothed with an 8mm FWHM isotropic Gaussian kernel to allow for residual variability after spatial normalization and the application of Gaussian Random Field Theory for corrected statistical inference. To remove low-frequency drifts, the data were high-pass filtered using a set of discrete cosine functions with a cut-off period of 128s.

Supplemental references

Abel S, Dressel K, Bitzer R, Kümmerer D, Mader I, Weiller C, *et al*. The separation of processing stages in a lexical interference functional MRI-paradigm. Neuroimage 2009; 44: 1113-24.

Blesser B. Speech perception under conditions of spectral transformation. I. Phonetic characteristics. J Speech Hear Res 1972; 15:5-41.

de Zubicaray GI, McMahon KL. Auditory context effects in picture naming investigated with event-related functional MRI. Cogn Affect Behav Neurosci 2009; 9: 260-9.

Henson RN. Efficient experimental design for functional MRI. In: Friston K, Ashburner J, Kiebel S, Nichols T, Penny W, editors. Statistical parametric mapping: The analysis of functional brain images. London: Elsevier; 2006. *P* . 193-210.

Hutton C, Bork A, Josephs O, Deichmann R, Ashburner J, Turner R. Image distortion correction in functional MRI: A quantitative evaluation. Neuroimage 2002; 16: 217-40.

Meyer AS, Schriefers H. Phonological facilitation in picture-word interference experiments: Effects of stimulus onset asynchrony and types of interfering stimuli. Journal of Experimental Psychology: Learning, Memory, and Cognition 1991; 17: 1146-60.

Schriefers H. Lexical and conceptual factors in the naming of relations. Cogn Psychol 1990; 22: 111-42.

Seghier ML, Ramlackhansingh A, Crinion J, Leff AP, Price CJ. Lesion identification using unified segmentation-normalisation models and fuzzy clustering. Neuroimage 2008; 41: 1253-66.

Shannon RV, Zeng FG, Kamath V, Wygonski J, Ekelid M. Speech recognition with primarily temporal cues. Science 1995; 270: 303-4.

Supplemental tables

Patient ID	Lesion locations	L IFC
P1	cortico-subcortical, occipito-parietal, posterior superior/middle temporal, posterior inferior parietal	spared
P2	cortico-subcortical, superior/middle/inferior temporal, posterior inferior parietal	spared
P3	cortico-subcortical, anterior parietal, frontal (ventrolateral, dorsolateral, dorsomedial), insular, basal ganglia	partly spared
P4	cortico-subcortical, superior/middle temporal, anterior inferior parietal, frontal (perirolandic, ventrolateral, dorsolateral), insular, basal ganglia	partly spared
P5	cortico-subcortical, occipito-parietal, posterior superior/middle temporal, posterior parietal	spared
P6	cortico-subcortical, occipito-parietal, posterior superior/middle temporal, posterior inferior parietal	spared
P7	cortico-subcortical, anterior superior temporal, frontal (ventrolateral), insular, basal ganglia	partly spared
P8	cortico-subcortical, superior/middle/inferior temporal, inferior anterior parietal	spared
P9	cortico-subcortical, occipito-temporal, superior/middle/inferior temporal, inferior parietal, frontal (ventrolateral, dorsolateral), insular, basal ganglia	partly spared
P10	cortico-subcortical, perisylvian, superior temporal, frontal (perirolandic, ventrolateral), insular, basal ganglia	partly spared
P11	cortico-subcortical, perisylvian, superior temporal, anterior inferior parietal, frontal (perirolandic, ventrolateral), insular, basal ganglia	partly spared
P12	cortico-subcortical, perisylvian, superior/middle temporal, anterior parietal, frontal (perirolandic, ventrolateral, dorsolateral), insular, basal ganglia	partly spared
P13	cortico-subcortical, perisylvian, superior temporal, frontal (perirolandic, ventrolateral), insular, basal ganglia	partly spared
P14	cortico-subcortical, anterior superior/middle temporal, frontal (perirolandic, ventrolateral), insular, basal ganglia	partly spared
P15	cortico-subcortical, perisylvian, perirolandic, superior/middle temporal, anterior inferior parietal, insular, basal ganglia	spared
P16	cortico-subcortical, perisylvian, superior/middle temporal, inferior parietal, frontal (perirolandic, ventrolateral, dorsolateral), insular	partly spared
P17	cortico-subcortical, occipito-parietal, superior/middle temporal, inferior superior parietal	spared
P18	cortico-subcortical, occipito-parietal, perisylvian, superior/middle/inferior temporal, inferior parietal, frontal (perirolandic), insular	spared

Supplemental Table 1 - Qualitative description of lesion locations on a subject-by-subject basis. Legend: L IFC = left inferior frontal cortex (whether spared or partly spared).

	Accuracy (%)						RT (ms)					
	T1		T2		T3		T1		T2		T3	
Patient ID	UNT	TRE#	UNT	TRE	UNT	TRE	UNT	TRE#	UNT	TRE	UNT	TRE
P1	62	63	63	90	64	83	1336	1297	1292	1213	1277	1261
P2	30	31	32	46	36	48	1626	1632	1559	1575	1698	1893
P3	22	28	34	37	39	39	2007	1936	1802	1732	1648	1591
P4	32	37	34	60	38	49	1601	1612	1759	1389	1598	1481
P5	55	57	68	87	70	85	1486	1502	1505	1342	1205	1131
P6	64	61	70	81	68	84	1573	1574	1462	1259	1612	1459
P7	66	85	75	82	68	81	1216	1192	1136	1073	1256	1233
P8	42	39	37	75	45	61	1630	1663	1385	1247	1384	1366
P9	52	53	55	79	57	71	1315	1330	1234	1215	1551	1282
P10	38	35	54	87	58	76	1706	1658	1082	933	1215	1140
P11	55	57	73	80	66	79	1632	1617	1421	1306	1684	1623
P12	22	24	29	63	33	51	1597	1684	1316	1221	1409	1310
P13	0	1	3	5	0	3	1142	1142	1477	1237	1451	1120
P14	54	49	55	85	54	71	1520	1529	1455	1236	1309	1324
P15	65	61	69	93	71	87	1555	1517	1699	1315	1718	1620
P16	41	40	32	75	42	74	1540	1590	1475	1160	1461	1416
P17	26	18	27	71	24	52	1008	1085	1200	1138	1259	1194
P18	36	34	44	96	66	91	1446	1617	1488	1038	1395	1151
mean (s.d.)	***42 (18)***	***43 (20)***	***47 (20)***	***72 (23)***	***50 (19)***	***66 (22)***	***1496 (229)***	***1510 (218)***	***1430 (201)***	***1257 (184)***	***1452 (177)***	***1366 (212)***

Supplemental Table 2 - Individual patients' raw scores at each time point of Experiment 1. Legend: RT = reaction time; ms = milliseconds; T1 = pre-treatment measure; T2 = post-treatment measure; T3

= follow-up (at three months); UNT = untreated items; TRE = treated items (at T1, TRE# = 'to-be-treated' items).

		Accuracy		RT	
		**T2-T1**	**T3-T1**	**T2-T1**	**T3-T1**
age	r	-0.28	-0.31	-0.33	-0.17
	P	0.268	0.204	0.183	0.512
months post-stroke	r	-0.25	-0.25	-0.11	-0.18
	P	0.314	0.308	0.669	0.474
hours of therapy	r	0.25	0.11	0.37	0.36
	P	0.326	0.657	0.132	0.147
lesion volume	r	0.00	-0.06	-0.07	0.24
	P	0.997	0.806	0.795	0.339

Supplemental Table 3 - Correlations between indexes of treatment outcome and demographic/clinical data. Indexes of treatment outcome have been computed as differences between treated items at different time points (as specified in the columns). Legend: RT = reaction time; T1 = pre-treatment measure; T2 = post-treatment measure; T3 = follow-up (at three months); r = Pearson's correlation coefficient; *P* = associated *P* -value.
